# Average or extraordinary? A tale of two studied samples’ anxiety related recovery work after COVID-19

**DOI:** 10.3389/fpubh.2025.1626124

**Published:** 2025-09-24

**Authors:** Gail Low, Alex Bacadini Franca, Anila Naz, Gloria Gutman, Zhiwei Gao, Sofia Von Humboldt

**Affiliations:** ^1^Faculty of Nursing, MacEwan University, Edmonton, AB, Canada; ^2^Laboratory of Human Development and Cognition, Federal University of São Carlos, São Paulo, Brazil; ^3^Faculty of Nursing, University of Alberta, Edmonton, AB, Canada; ^4^Department of Gerontology, Simon Fraser University, Vancouver, BC, Canada; ^5^Faculty of Medicine, Memorial University, St. John’s, NL, Canada; ^6^William James Center for Research, Lisbon, Portugal

**Keywords:** network analyses, anxiety management, convenience sample, representative sample, flourishing and positive and negative feelings

## Abstract

**Introduction:**

A global pandemic is a hardship and mentally distressing event for any of us, and particularly for people living at a greater risk of post-infectious health harms. Public discourse about COVID-19 largely characterizes older people as a physically and mentally vulnerable demographic. Research findings largely now to the contrary consider age an asset, a perspective in keeping with Seligman’s idea that everyday people can also see the positive side of life and act accordingly when faced with events that are neither positive nor within their control. With this in mind, we explore how average older people were managing pandemic-related anxiety when mandated COVID-19 public health measures were lifted.

**Methods:**

Our primary study sample was a national census-based quota sample (*N* = 1,327) of average older Canadian people. A second study sample was recruited by convenience (*N* = 1,200) for comparison purposes. Both groups responded to an e-survey launched between July 1st and up to August 16th, 2022, about how anxious they felt and how they were managing at this key turning point.

**Results:**

Convenience sample responders were largely residing in Ontario (*Z* = 781.667, *p* < 0.001), in very good to excellent health (*Z* = 180.534, *p* < 0.001), and university educated (*Z* = 1285.255, *p* < 0.001). Far fewer were in their 60s (*Z* = 124.898, *p* < 0.001; *Z* = 22.349, *p* < 0.001). Descriptive network analyses revealed that the two studied samples had in common a diverse and purposive network of coping strategies for managing pandemic-related anxiety.

**Discussion:**

Average older Canadians managed their anxiety as capably as healthier, better educated, and generally older peers. Our findings are explored through a lens of positivity, not vulnerability. Methodological provocations are offered for future research, including post-pandemic between-sampling comparisons.

## 1 Introduction

Seligman has long argued that the human mindset during unavoidable, perilous events is not one of giving up and giving way (1, 2). People also have the capacity to see them as neither permanent nor beyond their capacity to deal with. Everyday people can and do see “the positive side of life” (2, p. 231) and so do positive things. They might engage in activities that ideally hold them captive or find some sense of meaning through being of service to individuals or to contribute to a larger cause. What each element looks like is determined by the person living that life. In theory, people are also motivated to accomplish things, to get things done and as best as they can, regardless of whether their acting on these motives benefits other aspects of their well-being such as their relationships with other people, their health, or their capacity for positive emotions (1, 2).

Research studies before COVID-19 speak to older people’s capacity to see the positive side of life. Older Japanese people embracing rather than fearing what the future holds were seemingly staving off losses in their physical and mental functioning over four years of time (3). Older Turkish people who were doing things that are meaningful and gave them a sense of purpose appeared to be less prone to depression (4). Older Spaniards more inclined to see opportunities for own-growth and to have a sense of purpose in life (5) were reporting lesser symptoms of anxiety. Norwegian contemporaries taking part in activities when they wanted to (6) and Thai older adults doing what they wanted to the best of their ability (7) appeared to stave off depression and anxiety. Some older German people believing that they were actively contributing to society in some way reported lower depressive symptoms (8). A meta-analysis across 10 countries revealed that older people with similar mindsets were generally better able to persevere through adversity (9).

A pandemic can put anyone’s mental resolve to the test and is fertile ground for learning what everyday people in situations beyond their control are capable of (10). In a pandemic like COVID-19, people live with very real threats to their health and safety, connectedness to others, and paid and/or unpaid occupations (11). Early on in the pandemic, it was public knowledge that it was largely older Canadians (12) and older people abroad (13) who were dying from COVID-19. Older Canadians were also being stereotyped as vulnerable in outlets such as government documents (14) and newspapers (15). Aligning with Seligman’s sentiments (1, 2) however were older Canadians’ statistically significantly lesser likelihood of perceiving COVID-19 as a negative event (16) and fewer symptoms of depression (17) than midlife adults, and lesser anxiety symptoms (18) as also compared to younger adult counterparts. Similar patterns were reported among older Americans (19) and Brits (20). Older Canadians’ capacity to take action to manage a wide range of anxiety with more success than indifference led to their being characterized as perseverant (21). These findings support others’ observing that as people get older, they can also get better at regulating negative emotions (22–24).

Seligman theorizes that people’s seeking to be of service to others is not contingent upon it being self-beneficial (1, 2). Recent research also tells us that some older people find some semblance of meaning in being of service to others, and perhaps even better health and relationships with others. Older people in Czechoslovakia, Germany and Hong Kong were putting their knowledge and skills to use through volunteering and mentoring simply because it was a meaningful thing for them to do (25). Across 16 European countries, non-partisan volunteers stepping out of their comfort zones were also learning new things and trusting others again (26). Older Brazilians were reporting higher mental health than self-oriented age-peers, and over one year of time (27). Self-worth among Canadians in their 50s has been linked to opportunities for setting a good example for others and passing on the benefits of their life experiences (28). Serving communities in some capacity can have snowball effects on older people’s lives (29). Volunteering has given some 13,000 older Americans a greater sense of purpose in life over 4 years of time (30).

Mayr and colleagues also point out that everyday older people who engage in work for others’ benefit likely do so from a genuinely good place (31). Before COVID-19 and across 16 countries, being a volunteer or a mentor in later life was also associated with emotional fulfillment, and more so than one’s own notoriety (32). Among some 109,000 Asian and African older adults, a desire to help others in need with everyday tasks and providing emotional support was negligibly associated with social status, including how wealthy or educated you are (33). For some older Italians, happiness was not statistically significantly associated with putting yourself first, but rather with helping others and seeing someone other than yourself prosper (34).

Well-intentioned older people also want to do things that make a difference in others’ lives in the here-and-now (35). During the first year of COVID-19, some older Canadian women equated being happy with doing something good for others now, such as picking up the phone rather than waiting for them to call you (36). Focusing on getting by one day at a time helped Americans in their early 60s feel less mentally distressed when COVID-19 lockdowns ended (37). When physical distancing ended, older people across Canada advised age-peers to focus on what they could do to safely re-enter open spaces, not what they would not be able to do if they caught COVID-19 (38).

The broader message from this literature is, as Seligman (1, 2) believes, that everyday people can see the positive side of life and to act accordingly, such as through being of service to others (1, 2). Some older people have reported finding some sense of meaning, chances to get to know others, and perhaps even less anxiety. In our eyes, older people serving their communities in some capacities are also doing things that defy vulnerability stereotyping. Older people are not all the same; they contribute to society through a variety of activities and for the collective good (39). After public health measures were lifted, older Canadians shared social isolation remedies for peers nationwide and for a knowledge mobility product, despite a good number feeling severely anxious (38). This past year, Taiwanese people seeing themselves as influencers, positively emotionally connected to people and to places across 17 districts, reported living more satisfying lives (40). People who seek out opportunities to do and to see what they are capable of, albeit as a volunteer or a companion, position themselves for better mental health (41).

Pandemics can limit and interfere with how any of us typically serve our communities, such as through mandated public health measures, and can be anxiety provoking (11). This study explores pandemic-related anxiety management among two groups of older people, when mandated COVID-19 public health measures were lifted. With this in mind, we use network analyses to describe how average older people were managing their pandemic-related anxiety. Variable systems or networks of nodes and links can help researchers describe mechanisms behind how people may go about protecting their mental health (42). A second network analysis among a generally older, healthier, and more educated peer group gave us some context within which to better interpret average older Canadians’ observed coping strategy networks.

## 2 Materials and methods

### 2.1 Study design and procedure

A cross-sectional design was conducted across 10 Canadian provinces. The study was carried out by the co-investigators, in partnership with Qualtrics, a formidable, global e-survey company (43).

### 2.2 Participants

National Sample (NS) and Convenience Sample (CS) inclusion criteria were: (1) individuals who were residing in any Canadian province and (2) at least 60 years of age.

### 2.3 Data collection

Our cross-sectional e-survey work began on July 1st, 2022. To recruit our NS, Qualtrics sent a study advertisement (Supplementary Appendix S1) to its Consumer Panel Members residing across all 10 Canadian provinces. Potential responders are asked to help us learn about how they were managing pandemic-related anxiety and for creating a mental health cookbook-style recipe book.

Stratified random sampling was used and based on the most recently available age, sex, and education census distributions (44). Canadian Census proportions for 2021, which also included gender identity, were not yet available. We knew the proportion of LGBTQ2 + older Canadians as a whole per se, as disclosed for public consumption by Statistics Canada (45).

Potential NS participants responding to the advertisement were taken to the study information and an informed consent section on a Qualtrics landing page (Supplementary Appendix S2). To protect confidentiality and further enhance data quality, those indicating ‘Yes’ to taking part in our study were assigned a unique identifier number and a single-use link to prevent multiple completions. Fortunately, the Qualtrics platform also supports bot detection. Data was collected until August 15th, when responses stopped completely. We received a scrubbed dataset from Qualtrics on August 16th.

Our CS was recruited through an August 2nd email blast sent by the RTOERO (46) to its members at large, using the same advertisement. Because we had no way of knowing whether RTOERO members were also Consumer Panel Members, their study information and an informed consent letter (Supplementary Appendix S3) reflect this. Interested responders clicking on the hyperlink embedded in the advertisement were taken to a separate Qualtrics e-survey landing page. ‘Yes’ responders were assigned a unique password and a single-use e-survey link. Our budget permitted a responder quota of *n* = 1,200 was met within 24 h. Qualtrics sent us the corresponding dataset on August 16th.

### 2.4 Scale

The study used the Geriatric Anxiety Scale 10 (47), a short-form questionnaire found to be reliable and valid among community-dwelling older people (48, 49). In this study, the GAS-10 exhibited excellent internal consistency reliability in our national sample (*α* = 0.921; *ω* = 0.924) and our convenience sample (*α* = 0.890, *ω* = 0.890).

Older Canadians self-identified coping strategies to manage their pandemic-related anxiety using the Centre for Addiction and Mental Health Coping with Stress and Anxiety checklist (50) (*α* = 0.747, *ω* = 0.751 for the national sample; *α* = 0.680, *ω* = 0.683 for the convenience sample). This personal Coping with Stress and Anxiety checklist for public consumption was made available as social distancing was lifted and still is.

Both responder groups were asked to share their age, sex-at-birth, gender identity, education level, marital status, home province, and perceived health and numbers of chronic illnesses.

## 3 Statistical analysis

Univariate statistics were generated for participants’ self-identified personal and health characteristics, anxiety levels, and coping strategies using SPSS V27.0. Statistically significant between-group differences across all such categorical variables were identified using Bootstrap for Independent-Samples Proportions Tests (51), with Phi as a measure of effect size (52). Network analyses were conducted using R Version 3.3.1 with the bootnet, ggraph, and NetworkComparisonTest packages. We wanted to describe and compare networks of strategies for managing pandemic-related anxiety between average and seemingly advantaged older Canadians.

### 3.1 Network estimations

We estimated partial correlation networks for both the National Sample (NS) and the Convenience Sample (CS) using the IsingFit method (53), which is designed for binary data. Their thickness reflects the magnitude of the regularized partial correlations between pairs of nodes. All items were dichotomous and did not require transformation. Network estimation was performed using the graphical least absolute shrinkage and selection operator (Glasso), with model selection guided by the Extended Bayesian Information Criterion (EBIC) and a default hyper parameter gamma of 0.50 (54, 55). Participants with missing responses were removed via list wise deletion. The final network estimation included only complete cases. Items were categorized *a priori* into two conceptual domains — anxiety symptoms and coping strategies.

### 3.2 Network visualizations

The resulting networks were visualized using the qgraph package (56), wherein network nodes represent an instrument’s items, and their edges depict their relationships, with edge weights reflecting the strength of associations between them, all things being considered. However, as is common in the literature, we concentrated our interpretation on the most central nodes rather than on specific edge strengths, as this approach is widely adopted in empirical studies.

### 3.3 Centrality measures

We calculated centrality indices using standardized values (57). We examined strength centrality, which represents the sum of all edge weights connected to a node. Higher values indicate greater influence within a network. Results were visualized using z-score standardization.

### 3.4 Stability and bootstrap analysis

We assessed the robustness of the estimated network structures using a case-dropping bootstrap procedure (58). Stability was quantified using the CS-coefficient, which determines the maximum proportion of cases that can be dropped while retaining accuracy with 95% confidence. The recommended cut-off point for the CS-coefficient is 0.25, values above 0.50 are preferable (59).

### 3.5 Network comparison test

Network Comparison Tests (NCT) were employed (60) to evaluate structural differences between the NS and CS networks. We paid particular attention to between-network differences in global network structure invariance, edge-specific strength, and overall connectivity (global strength). All such tests were conducted using 1,000 permutations, with edge-specific comparisons performed when applicable (60).

### 3.6 Ethical aspects

The study received renewal approval from the Ethics Committee of the Faculty of Nursing, University of Alberta, Pro00118512 (March 9, 2022), REN_2 (December 18, 2023), REN_3 (December 17, 2024) and now MacEwan University (File No. 102407, November 7, 2024; January 8, 2025). The study adhered to the principles outlined in the Helsinki Declaration.

## 4 Findings

### 4.1 Study participant characteristics, anxiety levels, and coping strategies

Responders in the CS expectedly differed from NS responders (see Table 1). Convenience sample responders were largely university educated (*X*^2^ = 1285.255, *p* < 0.001, *φ* = 0.71, strong effect) and resided in Ontario (*X*^2^ = 781.667, *p* < 0.001, *φ* = 0.56, strong effect), in very good to excellent health (*X*^2^ = 180.53, *p* < 0.001, *φ* = 0.27, small effect). Unlike our NS responders, none of our CS responders reported having no degree, diploma, or certificate (*X*^2^ = 277.086, *p* < 0.001, *φ* = 0.33, moderate effect). Far more CS responders were born female (*X*^2^ = 137.677, *p* < 0.001, *φ* = 0.24, small effect) and self-identified as such (*X*^2^ = 81.812, *p* < 0.001, *φ* = 0.20, small effect), and far fewer were in their 60s (*X*^2^ = 124.898, *p* < 0.001, *φ =* 0.22, small effect).

**Table 1:** Personal and health characteristics for national and convenience sample participants.

Characteristics	National sample (*n* = 1,327) *n* (%)	Convenience sample (*n* = 1,200) *n* (%)	*X* ^2^	*p-*value	Effect size (Phi – φ)
Age (years)					
60–64	384 (29.1%)	127 (10.9%)	124.898	<0.001	0.22
65–69	335 (25.4%)	204 (17.5%)	22.349	<0.001	0.09
70–74	264 (20.0%)	319 (27.4%)	18.992	<0.001	0.08
75–79	131 (9.9%)	271 (23.3%)	81.543	<0.001	0.18
80–84	145 (11.0%)	156 (13.4%)	3.423	0.064	0.03
85 years of age and older	61 (4.6%)	86 (7.4%)	8.539	0.003	0.05
Sex					
Female	671 (52.0%)	821 (75.3%)	137.677	<0.001	0.24
Male	620 (48.0%)	269 (24.7%)	−137.677	<0.001	0.24
Gender					
Cisgender woman	502 (42.0%)	492 (62.8%)	81.812	<0.001	0.20
Cisgender man	516 (43.1%)	202 (25.8%)	61.880	<0.001	0.17
Trans/non-binary	178 (14.9%)	90 (11.5%)	4.687	0.03	0.04
Chronic illnesses					
None	507 (39.2%)	485 (41.6%)	1.529	0.216	0.02
1	358 (27.7%)	364 (31.2%)	3.785	0.052	0.03
2	281 (21.7%)	216 (18.5%)	3.832	0.050	0.03
3 or more	148 (11.4%)	100 (11.4%)	5.506	0.019	0.04
Perceived health					
Poor/fair	407 (31.1%)	162 (13.8%)	105.003	<0.001	0.20
Good	604 (46.1%)	443 (37.7%)	18.089	<0.001	0.08
Very good/excellent	298 (22.8%)	570 (48.5%)	180.534	<0.001	0.27
Education level					
University certificate, diploma or degree below, at, or above Bachelor’s	275 (20.8%)	1,064 (89.6%)	1285.255	<0.001	0.71
College, CEGEP, or other non-university certificate or diploma	238 (18.0%)	93 (7.8%)	15.888	<0.001	0.08
Apprentice, Trades certificate or diploma	168 (12.7%)	8 (0.7%)	391.546	<0.001	0.39
High school/equivalency certificate	392 (29.6%)	23 (1.9%)	186.689	<0.001	0.27
No certificate, diploma, or degree	251 (19.0%)	0 (0%)	277.086	<0.001	0.33
Province					
British Columbia	226 (17.8%)	41 (3.5%)	130.286	<0.001	0.23
Prairie Provinces	254 (20.0%)	11 (0.9%)	231.993	<0.001	0.30
Ontario	510 (40.3%)	1,011 (93.8%)	781.667	<0.001	0.56
Quebec	148 (11.7%)	10 (0.8%)	119.177	<0.001	0.22
Maritime Provinces	129 (10.2%)	12 (1.0%)	94.884	<0.001	0.19

Chi-square (X^2^) was used to compare categorical variables between participants. Frequencies reflect missing responses.

There were some differences between our two studied samples when it came to their anxiety levels. Far fewer CS responders self-identified as having no anxiety at (*X*^2^ = 34.813, *p* < 0.001, *φ* ***=*** 0.17, small effect) and mild anxiety (*X*^2^ = 14.920, *p* < 0.001, *φ* = 0.07, small effect). And while NS responders were far less likely to report severe anxiety (*X*^2^ = 5.599, *p* < 0.001, *φ* ***=*** 0.01 small effect), this was the smallest observed statistically significant difference between them (Table 2).

**Table 2:** Anxiety scores for national and convenience sample participants.

Anxiety score category	National sample (*n* = 1,327) *n* (%)	Convenience sample (*n* = 1,200) *n* (*%*)	*X* ^2^	*p*-value	Effect size (Phi - φ)
Not at all anxious	273 (66.3%)	139 (33.7%)	34.813	<0.001	0.17
Minimal anxiety	719 (46.6%)	825 (53.4%)	69.556	<0.001	0.12
Mildly anxious	235 (62.2%)	171 (37.8%)	14.920	<0.001	0.07
Moderately anxious	32 (59.3%)	22 (40.7%)	0.646	0.421	0.04
Severely anxious	13 (86.7%)	2 (13.3%)	5.599	0.018	0.01

Chi-square test (X^2^) was used to compare categorical variables between participants. Frequencies reflect missing responses.

Responders in the CS were far more inclined to use 15 anxiety management strategies (Table 3). They were most inclined to seek credible information (*X*^2^ = 161.739, *p* < 0.001, *φ* = 0.25, small effect) and support from loved ones (*X*^2^ = 133.487, *p* < 0.001, *φ* = 0.23, small effect), to moderate their caffeine intake (*X*^2^ = 81.902, *p* < 0.001, *φ* = 0.18, small effect), eating healthy (*X*^2^ = 72.403, *p* < 0.001, *φ* = 0.17, small effect). Convenience responders also had a penchant for challenging their anxious thoughts and worries (*X*^2^ = 65.007, *p* < 0.001, *φ* = 0.16, small effect) and for staying active (*X*^2^ = 62.174, *p* < 0.001, *φ* = 0.15, small effect). Nonetheless NS responders used these most popular strategies most often themselves to manage their own anxiety. Both groups also unplugged from electronics (21.2 and 32.6%, respectively) and practiced relaxation and meditation (38.7 and 48.5%, respectively) least often. And while NS responders were more likely to decrease other sources of stress in their lives (*X*^2^ = 23.698, *p* < 0.001), as is the case with all others, this effect size (*φ* = 0.05) was small. Overall, both studied samples tried all 16 coping strategies, albeit with varying frequency.

**Table 3:** Coping strategies for national and convenience sample participants.

Coping strategy	National sample (*n* = 1,327) *n* (*%*)	Convenience sample (*n* = 1,200) *n* (*%*)	*X* ^2^	*p-*value	Effect size (Phi - φ)
I accepted that some fear and anxiety was normal	1,163 (89.1%)	1,152 (97.1%)	59.281	<0.001	0.15
I sought credible information, i.e., WHO, Health Canada, Provincial Ministry of Health, Local Public Health Unit	581 (44.7%)	828 (70.1%)	161.739	<0.001	0.25
I found a balance by staying tuned in (open to new stories about COVID-19) but knowing when to take a breather	1,016 (77.9%)	1,049 (88.4%)	49.189	<0.001	0.14
I brought an intentional mindset to unplugging from electronic devices, including phones, tablets, and computers	281 (21.2%)	386 (32.6%)	38.189	<0.001	0.12
I dealt with problems in a structured	1,020 (78.4%)	993 (84.7%)	16.295	<0.001	0.08
I remembered that I am resilient and was careful with WHAT-IFs (questions)	989 (75.8%)	991 (84.3%)	27.268	<0.001	0.10
I challenged worries and anxious thoughts	841 (64.6%)	930 (79.3%)	65.007	<0.001	0.16
I decreased other sources of stress in my life	756 (58.2%)	737 (63.0%)	6.616	0.013	0.05
I practiced relaxation and meditation	503 (38.7%)	567 (48.5%)	23.698	<0.001	0.09
I sought support from loved ones	565 (43.5%)	781 (66.6%)	133.487	<0.001	0.23
I was kind to myself	1,107 (84.8%)	1,047 (89.5%)	11.866	<0.001	0.06
I ate healthy	950 (73.0%)	1,023 (86.8%)	72.403	<0.001	0.17
I avoided substance use, including smoking, vaping and alcohol	854 (65.6%)	869 (74.2%)	21.673	<0.001	0.09
I had a moderate caffeine intake	976 (74.8%)	1,042 (89.0%)	81.902	<0.001	0.18
I got proper rest and sleep	1,012 (77.7%)	970 (82.5%)	8.732	0.003	0.05
I stayed active	914 (70.1%)	983 (83.6%)	62.174	<0.001	0.15

Chi-square (X^2^) was used to compare categorical variables between participants. Frequencies reflect missing responses.

### 4.2 Network comparison tests for coping strategies

#### 4.2.1 Network visualization and predictability

**Figure 1:**
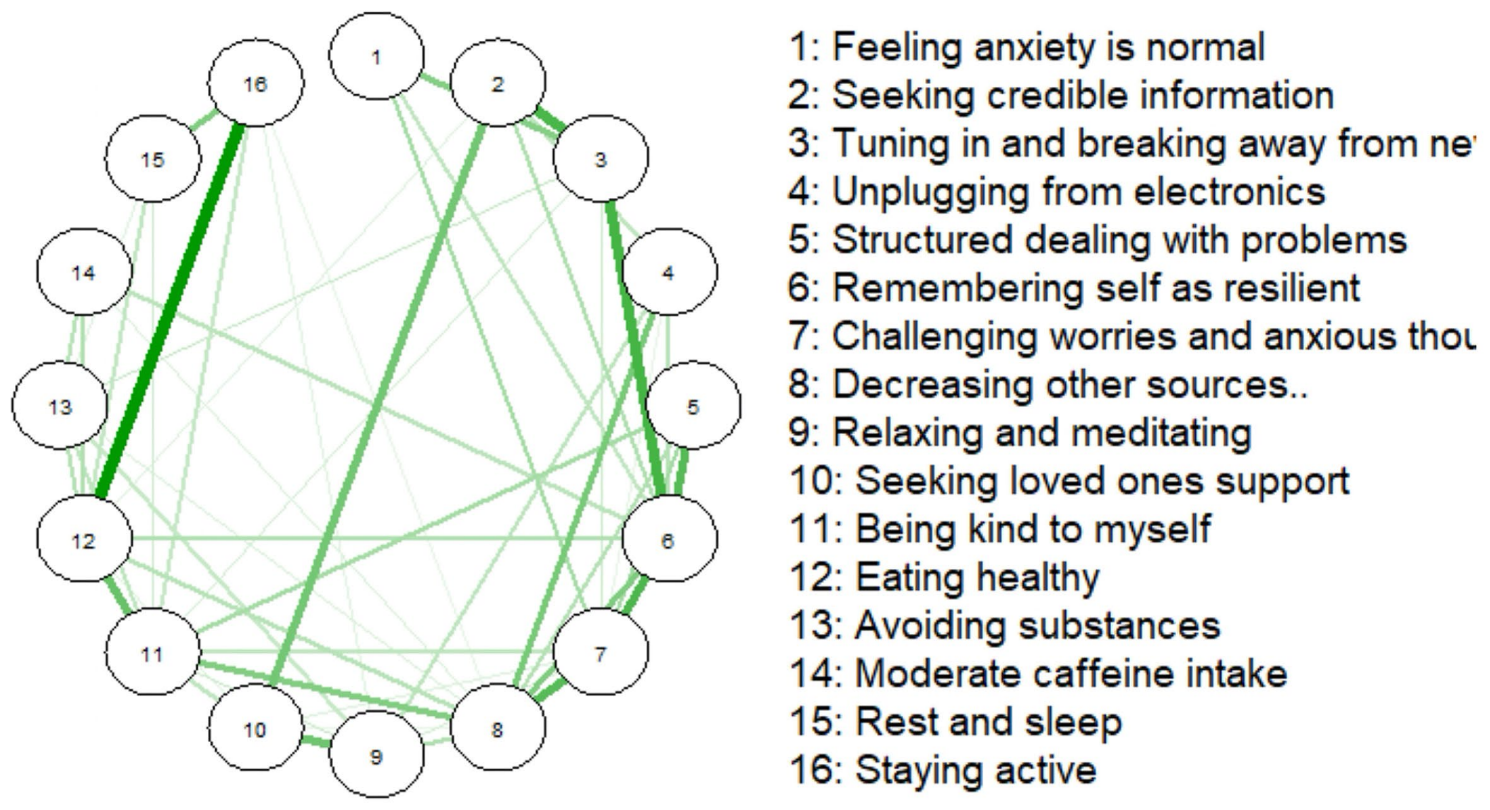
Coping behaviors network for the national sample (*n* = 1,327).

**Figure 2:**
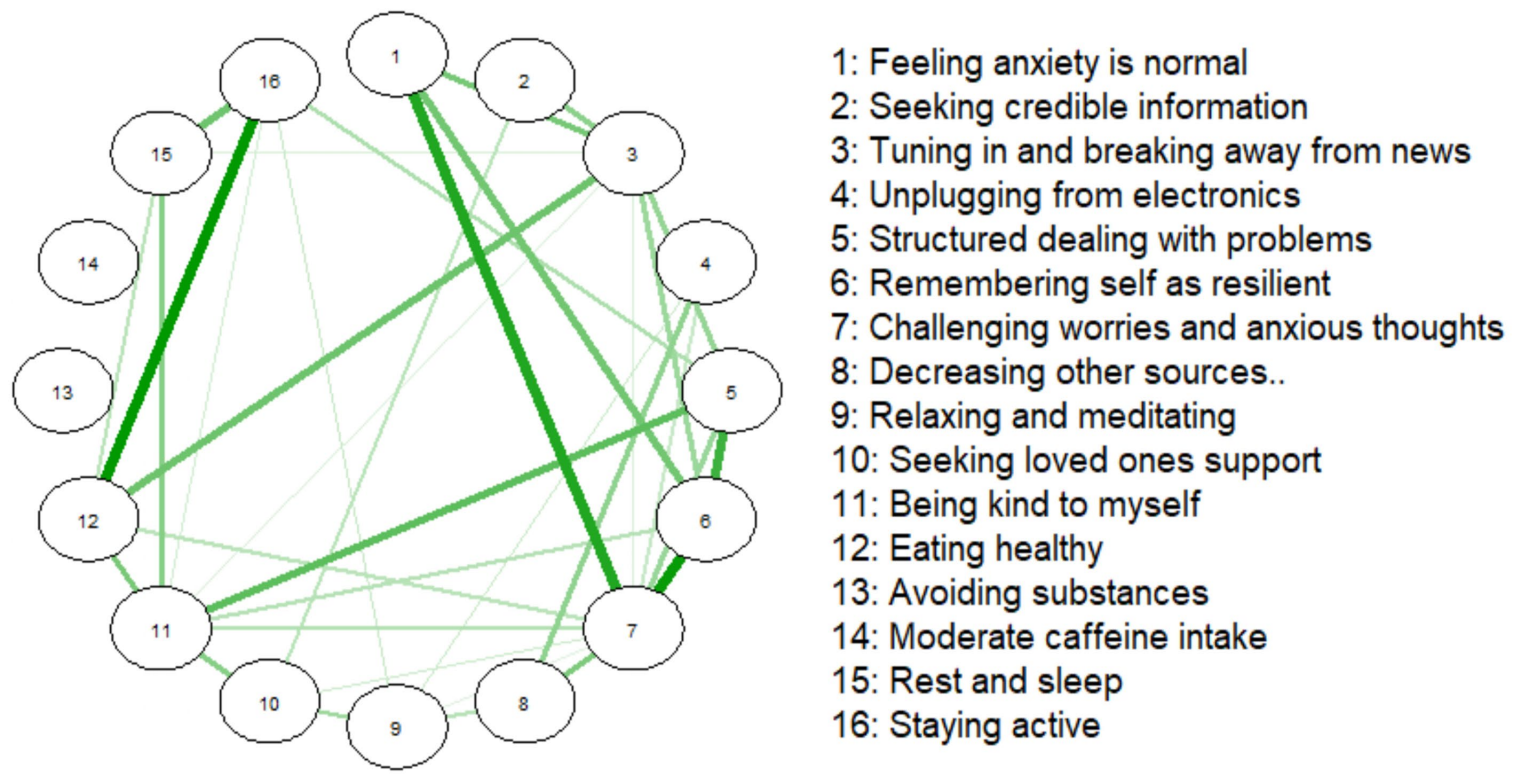
Coping behaviors networks for the convenience sample (*n* = 1,200).

Both networks are shown in tandem, as Figures 1, 2. The average predictability or the variance explained by neighboring nodes for both samples was 0.54. Remarkably, slightly more than half of each coping behavior’s variance could be explained by its direct connections to others.

#### 4.2.2 Edge-specific strength

The NS’s coping strategy network appeared denser, with stronger connections between certain strategies, the most being between ‘Staying active’ (16) and ‘Rest and sleep’ (15). Additionally, ‘Avoiding substances’ (13) and ‘Eating healthy’ (12) were strongly associated, with this perhaps indicating that health-conscious behaviors cluster together.

In the CS network, ‘Staying active’ (16) and ‘Rest and sleep’ (15) were also strongly associated, reinforcing our other observed link between physical activity and rest. The overall structure of the CS coping strategy network presented as more diffuse or weakly interconnected.

The NS network of coping strategies had a density of 0.433 (52/120 edges), with a mean edge weight of 0.194. The CS network was less dense (0.292; 35/120 edges), with a mean weight of 0.161.

#### 4.2.3 Centrality analysis

**Figure 3:**
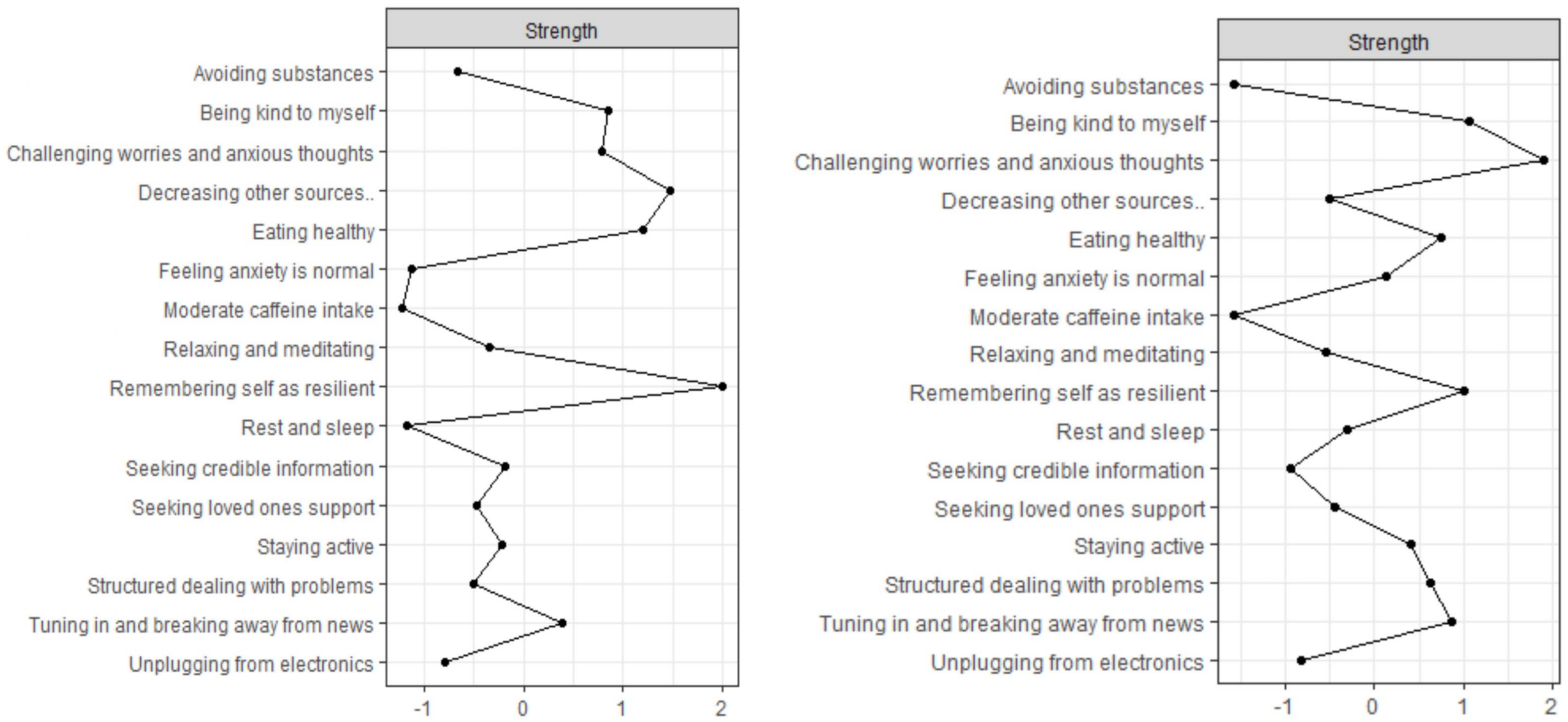
Coping behaviors networks for the national (*n* = 1,327; left-hand side) and convenience (*n* = 1,200) sample.

The centrality analysis of the coping strategy networks for the NS and CS revealed key differences in the relative importance of specific strategies within each group (Figure 3). Strength centrality indices were calculated to identify the most influential nodes in each network. In the NS, the nodes with the highest positive strength centrality were “Remembering self as resilient,” “Decreasing other sources,” and “Eating healthy.” In the CS, the most central nodes were “Challenging worries and anxious thoughts,” “Being kind to myself,” and “Remembering self as resilient.”

While resilience and maintaining healthy habits were central coping strategies among average older Canadians, challenging anxious thoughts figured most prominently among their seemingly more advantaged contemporaries. Despite these differences, “Remembering self as resilient” was among the top three most important strategies among both groups, highlighting its consistent importance for managing pandemic-related anxiety when public health measures were lifted.

### 4.3 Stability and bootstrap analysis

Stability, as indicated by the CS-coefficient for strength centrality, was deemed adequate in both samples, with values exceeding the recommended minimum threshold of 0.25. The CS-coefficient was 0.55 for the National Sample (NS) and 0.35 for the Convenience Sample (CS), suggesting sufficient stability for interpreting centrality estimates.

### 4.4 Global network structure invariance

The network invariance test revealed no statistically significant difference in the overall structure between the two networks (*M* = 0.824, *p* = 0.198). The overall configuration of coping strategies did not significantly differ between the two samples.

### 4.5 Global strength comparison

The global strength test tells us whether the overall connectivity (sum of edge weights) differed between the two studied samples. The results showed that the CS (24.049) coping strategy network exhibited a higher global strength (versus 20.875 in the NS). There was a tendency for stronger connections among coping strategies. However, this difference was not statistically significant (*S* = 3.174, *p* = 0.311). Coping strategies were organized in a relatively similar manner across both groups, without strong evidence of structural or connectivity differences.

## 5 Discussion

Our study is a descriptive network analysis of e-survey data collected from older Canadians when mandated COVID-19-related public health measures were lifted. While the COVID-19 pandemic has ended (61), there are lessons to be learned about what every day older people can be capable of when our mental resolve is put to the test. At the heart of any large-scale traumatic event like a pandemic, threats to our physical safety, particularly when self-relevant, frighten us most (62). During the COVID-19 pandemic, how old you were largely determined your risk for receiving urgent, intensive, and end-of-life care after catching COVID-19 (16). Older Canadians were categorized as most vulnerable to all such risks (17). They were most afraid of lingering physical threats even after the pandemic (63). However, in having practiced the lion’s share of COVID-19-related public health measures (64), they ended up being the least likely age group in Canada to be in physical harms-way (15).

Likewise, researchers in large-scale studies throughout the COVID-19 pandemic were deeming age a factor largely determining a person’s prospects for good mental health (19–23). Both CS and NS responders were using all 16 coping strategies, albeit variably frequently. NS responders were also using the very same strategies most often to manage pandemic-related anxiety. For example, both most often accepted that some fear and anxiety was normal or were kind to themselves. Just like CS responders, NS responders found parting from their electronic devices and relaxing and meditating least appealing. While challenging worries and anxious thoughts figured more prominently in the anxiety recovery work of CS responders and remembering that you are resilient was most central to NS responders, both groups undertook a wide array of anxiety recovery work. Challenging and remembering appeared to function as strategic stepping stones. Hence the greater presence of connections between challenging and remembering and many other coping strategies. For the NS, for example, remembering that you are resilient was connected with taking a break from COVID-19 news and dealing with problems in a structured way. For CS responders, challenging worries and anxious thoughts was connected to feeling that some anxiety was normal, and to remembered resilience. We saw more common than different recovery work, particularly with all coping strategy difference effect sizes being small.

All such common ground meant that NS responders’ self-identified networks of coping strategies were as predictable and as stable as CS responders’ networks. And while the strength of connections between strategies made NS responder networks appear denser, the connections between them were not remarkably stronger than CS responders’ network connections. The connection between staying active and getting enough sleep and rest is another case in point.

In having described and compared coping strategy networks among two different groups of older people to manage pandemic-related anxiety, we share some thoughts that could inform future research. First and foremost, everyday resources like advanced education enhance anyone’s capacity to earn money to be able to live in safe spaces and have enough food to eat (41). Education and income are things that largely determine our being in good health, particularly for marginalized groups (65). In Canada, older people have the lowest annual incomes (66) and are the most likely demographic to have less than secondary school education (67). Our NS responders were more likely to not have a diploma, degree or certificate (21% versus 0% in the CS). Differences in the proportion of CS (89.6%) versus NS (20.8%) responders reporting university education were large. NS responders were more likely to self-identify as being poor or fair and less likely to perceive their health as good to excellent, but to a much lesser extent. Despite these differences, NS responders fared as well as CS responders with respect to pandemic-related anxiety. While our CS responders were more likely to self-identify as being minimally anxious, a similar proportion of NS responders self-identified as mildly anxious, and far more of them reported having no anxiety at all. Our NS responders were significantly more prone to severe anxiety; however, this difference was the smallest observed difference.

With respect to managing anxiety, CS responders were significantly more likely than NS responders to seek support from loved ones. For both groups, however, seeking support from loved ones was connected with seeking credible information about COVID-19. This common ground makes sense given that authoritative health messaging is more often than not risk-elevating messaging that can heighten anyone’s anxiety (68, 69). Differences in either group’s propensity to seek support or credible information were small. Self-compassion seems to deter older people from gravitating toward unsavory coping behaviors like self-deprecation, and from negative emotions and mental stress (70). While being kind to yourself figured more prominently in CS responders’ anxiety management repertoires, across both groups, being kind was associated with dealing with problems in a structured way. Other older people who settle on dealing with problems as they surface, one at a time, report fewer symptoms of depression (71). Differences in either group’s propensity for being kind or dealing with problems in a structured way were very small.

Our findings as a whole suggest that NS responders were engaging in anxiety recovery work that was as diverse as a somewhat older and generally healthier, more educated group of peers. Observed differences in the central importance of “Remembering self as resilient” and “Challenging worries and anxious thoughts” were not sufficient enough to make the strength of connectedness or the overall configuration of either group’s coping strategy network remarkably different. One need only overlay the two networks to see such common ground. Observed differences in proneness to no anxiety at all or to severe anxiety between NS and CS responders were also small. Our NS responders’ observed capacity to manage pandemic-related anxiety was in keeping with a somewhat older, and generally healthier, more educated group of peers.

These findings have important implications for researchers inquiring about older people’s pandemic-related anxiety management, and for older people themselves. Mentally healthy recovery work in the aftermath of COVID-19 is likely as complex and long standing (72). While it is beyond the scope of a cross-sectional network analysis to infer cause and effect linkages between strategies (73), evidence of replication of how people cope during large-scale disasters can be compelling (5). A strength of this study is its comparison of the anxiety recovery work of two different groups of older Canadians, using the same survey data collected at the same key turning point in the pandemic.

Our study has limitations, however. Our comparison sample was recruited by convenience, through an e-blast. Convenience samples are systematically biased samples and researchers who primarily rely on them are said to end up with findings of limited external validity (74). Convenience sampling is also a timely and cost-effective method for learning about anxiety recovery work. Practically speaking, convenience sampling provided us with a feasible frame of reference in which to situate and to make sense of our NS findings. We were able to observe two different groups of older people adeptly managing anxiety within the context of a pandemic, and at a similar time.

Along with other older volunteers (25–27, 30, 32–34) and mentors (28, 32), average older Canadians in this study were sharing what managing anxiety looks like for them when mandated COVID-19 public health measures were lifted. Researchers studying people’s experiences across some 49 life years describe older people seeing whatever life throws at them as a chance to grow as flourishing (75). In contrast, negative emotions like anxiety can limit a person’s new experiences and ideas, which limit their social interactions and ability to acquire new skills and knowledge, negatively impacting overall well-being (76). So, to have a fulfilling and flourishing life it’s essential to experience more positive emotions and fewer negative ones. The presence of mental health characterized by high levels of emotional, social, and psychological well-being is considered a state of flourishing. Mental illness symptoms such as anxiety are inversely related to flourishing (77, 78). Some older Australians have conceived of the absence of hard-to-manage worries and anxieties as an element of flourishing (79). People who flourish are also said to be enterprising people working for the collective good (80, 81) using their resources at hand (31). Our somewhat younger, and less educated and healthy NS responders were also helping us to develop Cooking up calm, and to enhance public awareness in other ways such as through Global News, Renaissance Magazine, Good Times Magazine. However, because we did not directly measure flourishing, we cannot claim that our responders were. Any claims that we make here are purely interpretive and speculative.

Another important limitation concerns the lack of statistical control for demographic covariates such as age, education, and health status. The nationally representative and convenience samples differed substantially in these variables, which could partly explain observed differences observed in the network structures. Although widely used Ising model implementations and network comparison tools currently do not support covariate-adjusted estimation, a limitation noted in network methodology literature (82). Future studies could address this through matched subsamples, stratified analyses, or Bayesian approaches that allow for the inclusion of covariates (e.g., BGGM) (83). Until such methodologies become standardized in network science, interpretations of between-group differences should remain cautious and situated within the broader demographic context of each sample.

## 6 Conclusion

This descriptive network analysis study revealed that everyday older Canadian people were putting an equally broad repertoire of coping strategies to use at a particularly frightening time in the COVID-19 pandemic, and as predictably and as sufficiently stably as a somewhat older, and healthier and more educated group of peers.

## Data Availability

The datasets presented in this article are not readily available because there are ethical restrictions on the data being analyzed in this study. Questions about the ethical restrictions on sharing this data could be directed to REB@macewan.ca.
