# Supplementary material for: Average or extraordinary? A tale of two studied samples’ anxiety related recovery work after COVID-19
Source: Front Public Health. 2025 Sep 24;13:1626124. doi: 10.3389/fpubh.2025.1626124 (PMC12506091; doi:10.3389/fpubh.2025.1626124)
Supplement: Supplementary file 1 [file Supplementary_file_1.pdf]

Throughout the COVID-19 pandemic, Canadians 60+ years of age have been the most likely to get infected with this life-threatening virus, be hospitalized and need intensive care, and die from it. Since March 2020, when the pandemic was declared in Canada, social distancing has been necessary. For many older people, this has meant living alone at home in isolation, with moderate to severe anxiety, fear, and loneliness. Older Canadians have been the most at-risk for mental and physical harms in this pandemic. They are the most fearful (75% vs 63% of younger Canadians) of lingering permanent mental and physical harm after the pandemic ends. Yet, older Canadians are now needing to move out of their isolation from physically safer social spaces. Their mental health struggles could linger long after social distancing lifts and the COVID-19 pandemic ends.

We are working with a non-profit group known as the RTOERO Foundation. Our study aims are to identify mentally healthy living strategies that older Canadians have used in lock down and are using now to ease their way back into society, and also learn from them which strategies most reduce anxiety, stress, and other mental effects of the pandemic. We want to learn what has worked well for those who are thriving or doing well despite this pandemic. Identifying and learning this will lay the groundwork for creating a mental health cookbook-style recipe book.

We value your thoughts and opinion in this anonymous short survey. You are key to the success of this project. Please read the Study Information/Implied Consent Letter attached to this email first.

If you choose to take part in the survey, it will take you about 15-20 minutes to complete. The survey will close on: month, day, 2022 [4 months after the specific day when the survey is launched].

Should you have any questions or concerns about the study or the study survey, please contact the primary researcher at the University of Alberta Faculty of Nursing, Dr. Gail Low (email: gail.low@ualberta.ca; telephone: 780-492-8089).

Research team members:
Dr. Gail Low, Associate Professor, University of Alberta, Faculty of Nursing
Dr. Donna Wilson, Professor, University of Alberta, Faculty of Nursing
Dr. Gloria Gutman, Professor, Simon Fraser University, Department of Gerontology
Dr. Zhiwei Gao, Associate Professor, Memorial University, Faculty of Medicine Community Health and Humanities Division

Click Here to Complete Survey

UofA Ethics ID: Pro00118512
